# Infectious Mononucleosis Complicated with Bilateral Peritonsillar Abscess and Splenic Infarction

**DOI:** 10.1155/2021/6623834

**Published:** 2021-03-12

**Authors:** Mehrdad Hasibi, Mahsa Zargaran, Ali Asadollahi-Amin

**Affiliations:** Amir Alam Hospital, Tehran University of Medical Sciences, Tehran, Iran

## Abstract

Infectious mononucleosis (IM) due to Epstein–Barr virus (EBV) infection is usually self-limited. It presents with fever, pharyngitis, fatigue, and cervical lymph node enlargement. It is common among adolescents and young adults. Although most patients recovered without any sequelae, rare complications have been reported. We described a 28-year-old man with fever, sore throat, dysphagia, and a positive IgM viral capsid Ag (VCA Ag) for EBV infection. He was admitted and received dexamethasone. He developed bilateral peritonsillar abscess (PTA) and splenic infarction, rare complications of acute EBV infection, two days after discharge. Although early reports noted PTA might occur following dexamethasone administration, recently, no obvious evidence supports it. However, high erythrocyte sedimentation rate level in our patient might indicate bacterial superinfection, which could exacerbate with dexamethasone administration. Several mechanisms such as transient hypercoagulable state and insufficient blood supply due to splenomegaly were proposed for splenic infarction due to EBV infection. Since our patient remained asymptomatic during the disease, IM-associated splenic complications, including splenic infarction, should be kept in mind. Our patient underwent bilateral tonsillectomy and received conservative management for the splenic infarction. These two rare complications of acute EBV infection have not been reported simultaneously yet.

## 1. Introduction

Infectious mononucleosis (IM) consists of signs and symptoms usually associated with acute Epstein–Barr virus (EBV) infection. It is common among adolescents and young adults. It typically presents with fever, pharyngitis, fatigue, and cervical lymph node enlargement, which are self-limited. However, different complications have been reported in the course of the syndrome. In our patient, the disease course was complicated by bilateral peritonsillar abscess (PTA) and splenic infarction.

## 2. Case Report

On Nov 28th, 2020, a 28-year-old man was referred to our emergency department (ED) in Amir Alam Hospital, Tehran, with fever, sore throat, and dysphagia within the previous seven days. The patient had no significant medical history. He received intramuscular penicillin G followed by oral levofloxacin 500 mg/day without improvement before the admission.

His vital signs were noted for a temperature of 39.4°C, pulse rate of 105 beats/min, blood pressure of 110/60 mmHg, and respiratory rate of 18 per minute. Blood oxygen saturation on room air was 97%. He had bilateral tonsillar enlargement, covered by white exudation. Furthermore, bilateral and tender anterior cervical adenopathy was palpable. The abdominal examination revealed splenomegaly without tenderness. Other physical examinations were unremarkable. The result of complete blood count (CBC) showed 15,600/*μ*L leucocytes with 38% polymorphonuclears and 57% lymphocytes. The peripheral blood smear displayed 37% of atypical lymphocytosis without any blast cells. The liver function tests were within normal limits. The erythrocyte sedimentation rate (ESR) was 90 mm/hr, and C‐reactive protein (CRP) was 127 mg/L. Polymerase chain reaction (PCR) test of COVID-19 was negative. The blood culture was negative.

The patient was admitted with the diagnosis of infectious mononucleosis (IM). Acute EBV infection was confirmed by the positive IgM viral capsid Ag (VCA Ag). The patient's abdominal sonography showed splenomegaly.

Due to the severity of the patient's dysphagia and the high risk of upper airway obstruction, he received intravenous (IV) dexamethasone 8 mg twice a day. His condition improved partially after three days and was discharged from the hospital with oral prednisolone 30 mg/day.

Two days after discharge, the patient returned with high-grade fever, throat pain worsening, and dysphagia. Pharyngeal examination showed erythema and bilateral tonsillar swelling with whitish exudate. The ESR was 85 mm/hr.

**Figure 1:**
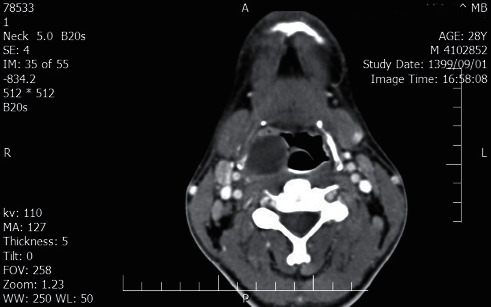
Head and neck CT scan with IV contrast shows right peritonsillar abscess (measuring 23 × 45 mm).

**Figure 2:**
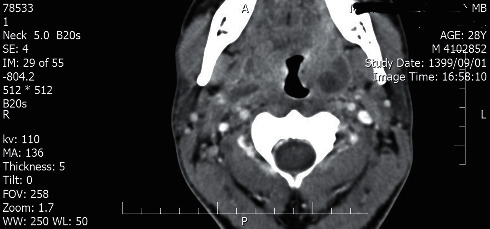
Head and neck CT scan with IV contrast shows left peritonsillar abscess (measuring 14 × 17 mm).

**Figure 3:**
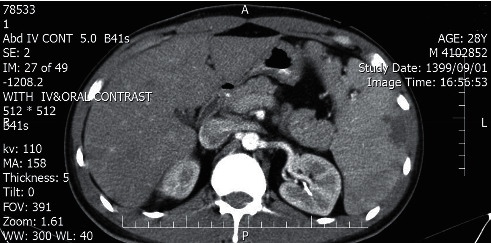
Abdominal CT scan with IV contrast shows splenic enlargement with a wedge-shaped, low-density defect in the peripheral area of the spleen in favor of splenic infarction.

The head and neck computed tomography (CT) scan with IV contrast demonstrated bilateral peritonsillar abscess (PTA) (measuring 23 × 45 mm on the right and 14 × 17 mm on the left side) (Figures 1 and 2). A chest and abdominal CT scan was done for ruling out lymphoma, which showed splenic enlargement with a wedge-shaped, low-density defect in the peripheral area of the spleen in favor of splenic infarction (Figure 3). The results of electrocardiography and transthoracic echocardiography were unremarkable.

Since the patient was treated with various antibiotics before the admission and the history of the first hospitalization, considering resistance organisms such as *Fusobacterium necrophorum*, IV piperacillin-tazobactam was administered. Also, a bilateral tonsillectomy was performed with no subsequent complications.

**Figure 4:**
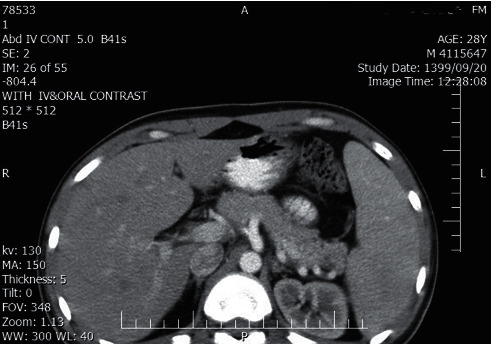
The splenic infarction resolves entirely in the following abdominal CT scan with IV contrast.

The histopathologic examination of the tonsils showed reactive follicular hyperplasia and abscess formation. Despite negative abscess culture results, IV antibiotic therapy was continued for seven days. As the splenic infarction was asymptomatic, it was managed conservatively. The patient was discharged by oral amoxicillin-clavulanic acid with the favorable condition and recommended to perform an abdominal CT scan two weeks later to follow up splenic infarction. The splenic infarction resolved entirely in the following abdominal CT scan (Figure 4).

## 3. Discussion

Infectious mononucleosis syndrome may be complicated by a wide range of complications such as upper airway obstruction, hepatosplenomegaly, and hemolytic anemia. PTA and splenic infarction are two rare complications of this syndrome [1–3].

PTA is reported in approximately 1% of the patients admitted with the diagnosis of IM [4]. The inflamed pharynx and necrotic tonsils in infectious mononucleosis are seldom complicated with bacterial superinfection such as hemolytic streptococcus (Lancefield groups A, C, and G), *Fusobacterium necrophorum*, and *Staphylococcus aureus* [1, 4]. Unilateral PTA usually presents with odynophagia, uvula, and soft plate deviation. In contrast, bilateral PTA is a rare complication and leads to no asymmetry or uvular deviation. An important differential diagnosis of bilateral PTA is tonsillar lymphoma, which may present with bilateral large and bulky tonsils [5]. In our case, lymphoma and leukemia were ruled out by the histopathology examination of tonsils and peripheral blood smear results.

During the first admission, our patient was treated with IV dexamethasone in the light of impending airway obstruction. The results of two previous studies showed that corticosteroid administration in patients with IM might be potentially associated with an increased risk of peritonsillar abscess formation [6, 7]. It should be mentioned that our patient had high ESR (90 mm/hr) in the first admission which is unusual in the setting of EBV infection. We presume that may be the patient had a superimposed bacterial infection at that time, which might exacerbate by IV corticosteroid administration. However, Hanna et al. did not find any evidence in favor of the IV corticosteroid role in PTA [6]. Unfortunately, we did not perform throat culture in the first admission to examine this hypothesis.

In the management of PTA, early surgical intervention is strongly recommended to fasten recovery and prevent deep neck space infection. Therefore, our patient underwent bilateral tonsillectomy [8, 9].

Several infection diseases could lead to a splenic infraction, such as babesiosis and malaria [10, 11]. Splenic infarction is a rare complication of infectious mononucleosis. Yan et al. found a total number of 23 cases of splenic infarction, complicated IM due to EBV, in the literature between 1961 and 2017 [12]. The exact mechanism of splenic infarction in IM remains unknown. It seems patients younger than 40 years of age are more likely to have an underlying hematologic illness, while patients over 40 years of age are more prone to suffer splenic infarction due to thromboembolic diseases [11, 13]. Insufficient blood supply due to hypercellular splenomegaly, especially in chronic hemolytic disorders, the transient hypercoagulable state, and increased levels of circulating immune complexes (CICs) which facilitate leukocyte aggregation and adhesiveness are noted as probable mechanisms for splenic infarction [12, 14]. In contrast to babesiosis, erythrocyte lysis by direct organism invasion is not a prominent mechanism of splenic infarction in IM; however, increased CIC levels may have a role in both diseases [10]. Similar to IM, the hypercoagulable state may contribute to splenic infarction in malaria. Furthermore, endothelial cell damage by malaria-infected red blood cells and malarial anemia are other proposed mechanisms [15]. Awareness of the diagnostic possibility of splenic infarction is crucial because it can lead to a splenic rupture with dramatic consequences and death. Splenic infarction may have a wide range of clinical presentations, similar to our patient; up to 30% of splenic infarction may be asymptomatic [16]. However, the most common clinical symptom is left-sided abdominal pain with left upper quadrant abdominal tenderness. Fever, vomiting, pleuritic chest pain, and pain referred to the back or shoulder are other possible signs and symptoms [11].

A previous study identified 163 adults at two South Carolina hospitals with splenic infarction based on clinical presentation and abdominal CT scan findings. Only 20% of these patients presented with classic left upper quadrant pain; 47% had abdominal pain elsewhere, and 33% had no abdominal pain at all. Notably, 40% of patients had more than one predisposing factor, including cardioembolism (25%), cancer (20%), sepsis (17%), inflammatory or infectious abdominal diseases (16%), and other conditions [17].

Although abdominal ultrasound (US) is considered as a diagnostic tool to detect splenic diseases (rupture, infarction, organomegaly, etc.), abdominal CT scan results revealed that the US is diagnostic only in 18% of patients [18, 19].

The treatment strategy for splenic infarction is primarily based on the underlying disease and ranges from supportive care to splenectomy [10, 20]. Life-threatening complications of splenic infarction are pseudocyst formation, abscess, hemorrhage, splenic rupture, and aneurysmal formation [20]. As our patient was asymptomatic and did not have any risk factors for splenectomy in babesiosis (hemoglobin of 10 mg/dl or less, platelet count of 50  ×  10⁹/L or less, presence of hemodynamic instability, and splenic rupture), mentioned by Dumic et al., conservative management strategy was selected [10]. And he was advised to avoid strenuous physical activity for 3–6 months, considering the chance of splenic rupture.

## 4. Conclusion

We describe a patient with IM who developed two rare complications, bilateral PTA and splenic infarction, simultaneously. Our management strategy was based on the patient's clinical symptoms and potential for life-threatening complications: tonsillectomy for bilateral tonsillar abscess and conservative approach for splenic infarction. Since our patient remained asymptomatic during the disease, IM-associated splenic complications, including splenic infarction and its fatal consequences, should be kept in mind.

## References

[1] Yaxley K. L. (2020). Infectious mononucleosis complicated by peritonsillar abscess and postural orthostatic tachycardia syndrome: a case report. *SAGE Open Medical Case Reports*.

[2] Ho K. M. A., Mitchell S. C. (2015). An unusual presentation of cardiac tamponade associated with Epstein-Barr virus infection. *BMJ Case Rep*.

[3] Heo D.-H., Baek D.-Y., Oh S.-M., Hwang J.-H., Lee C.-S., Hwang J.-H. (2017). Splenic infarction associated with acute infectious mononucleosis due to Epstein-Barr virus infection. *Journal of Medical Virology*.

[4] Johnsen T. (1981). Infectious mononucleosis and peritonsillar abscess. *The Journal of Laryngology & Otology*.

[5] Asad U., Warraich I., Idicula W. (2020). Infectious mononucleosis-related tonsillar hyperplasia mimicking T-cell lymphoma on histopathology: a rare case and review. *Acta Oto-Laryngologica Case Reports*.

[6] Hanna B. C., McMullan R., Hall S. J. (2004). Corticosteroids and peritonsillar abscess formation in infectious mononucleosis. *The Journal of Laryngology & Otology*.

[7] Handler S. D., Warren W. S. (1979). Peritonsillar abscess: a complication of corticosteroid treatment in infectious mononucleosis. *International Journal of Pediatric Otorhinolaryngology*.

[8] Alsubaie H. M., Alsmadi M. B., Aljuaid E. F. (2020). Bilateral peritonsillar abscess: a case study and literature review. *Journal of Surgical Case Reports*.

[9] Dalton R. E., Abedi E., Sismanis A. (1985). Bilateral peritonsillar abscesses and quinsy tonsillectomy. *Journal of the National Medical Association*.

[10] Dumic I., Madrid C., Rueda Prada L., Nordstrom C. W., Taweesedt P. T., Ramanan P. (2020). Splenic complications of babesia microti infection in humans: a systematic review. *Canadian Journal of Infectious Diseases and Medical Microbiology*.

[11] Naviglio S., Abate M. V., Chinello M., Ventura A. (2016). Splenic infarction in acute infectious mononucleosis. *The Journal of Emergency Medicine*.

[12] Yan L., Ann G., Sami A., Jennifer P. W., George M. A. (2018). Splenic infarction: an under-recognized complication of infectious mononucleosis?. *Open Forum Infect Dis*.

[13] Smalls N., Obirieze A., Ehanire I. (2015). The impact of coagulopathy on traumatic splenic injuries. *The American Journal of Surgery*.

[14] Suzuki Y., Kakisaka K., Kuroda H., Sasaki T., Takikawa Y. (2018). Splenic infarction associated with acute infectious mononucleosis. *The Korean Journal of Internal Medicine*.

[15] Hwang J.-H., Lee C.-S. (2014). Malaria-induced splenic infarction. *The American Journal of Tropical Medicine and Hygiene*.

[16] Noor M., Sadough M., Chan S., Singh G. (2017). Splenic infarct in a patient with infectious mononucleosis: a rare presentation. *Journal of Community Hospital Internal Medicine Perspectives*.

[17] Mueller P. S., Neda A., Emily M. M. (2020). Splenic infarction: clinical features and associated conditions. *JAMA Internal Medicine*.

[18] Antopolsky M., Hiller N., Salameh S., Goldshtein B., Stalnikowicz R. (2009). Splenic infarction: 10 years of experience. *The American Journal of Emergency Medicine*.

[19] Mackenzie D. C., Liebmann O. (2013). Identification of splenic infarction by emergency department ultrasound. *The Journal of Emergency Medicine*.

[20] Mamoun C., Houda F. (2018). Splenic infarction revealing infectious endocarditis in a pregnant woman: about a case and brief literature review. *Pan African Medical Journal*.

